# Dependence of Electron Density on Fermi Energy in N-Type Gallium Antimonide

**DOI:** 10.6028/jres.108.019

**Published:** 2003-06-01

**Authors:** Herbert S. Bennett, Howard Hung

**Affiliations:** National Institute of Standards and Technology, Gaithersburg, MD 20899-8120 USA

**Keywords:** band structure, dopants, electron density, Fermi energy, gallium antinomide, Raman measurements

## Abstract

The majority electron density as a function of the Fermi energy is calculated in zinc blende, n-type GaSb for donor densities between 10^16^ cm^−3^ and 10^19^ cm^−3^. These calculations solve the charge neutrality equation self-consistently for a four-band model (three conduction sub-bands at Γ, L, and X and one equivalent valence band at Γ) of GaSb. Our calculations assume parabolic densities of states and thus do not treat the density-of-states modifications due to high concentrations of dopants, many body effects, and non-parabolicity of the bands. Even with these assumptions, the results are important for interpreting optical measurements such as Raman measurements that are proposed as a nondestructive method for wafer acceptance tests.

## 1. Introduction

Most interpretations of optical measurements on compound semiconductors such as GaSb require physical models and associated input parameters that describe how carrier densities vary with dopant concentrations and measured Fermi energies. In this paper, we report on a method that gives closed form analytic expressions for the carrier densities in the conduction sub-bands for GaSb at room temperature. The method is based on an iterative and self-consistent solution of the charge neutrality equation with full Fermi-Dirac statistics for the carriers at finite temperature and on the use of statistical analyses to give analytic expressions that represent the calculated data sets.

The method reported here is related to earlier work on n-type GaAs presented in reference [1]. Reference [1] gives the results predicted by an effective two-band model, one equivalent conduction band and one equivalent valence band at Γ, that includes the densities of states modifications due to high concentrations of dopants and due to many-body effects associated with carrier-carrier interactions. The method given below for GaSb is a four-band model. But, because of computational limitations, it does not include the densities of states modifications due to high concentrations of dopants and due to many-body effects.

## 2. Theory

The electron *n* and hole concentrations *h* in units of cm^−3^ at thermal equilibrium are given, respectively, by

$$n=\int_{-\infty }^{+\infty }f_{0}(E)\rho_{c}(E)dE\;\text{and}\;h=\int_{-\infty }^{+\infty }[1-f_{0}(E)]\rho_{v}(E)dE,$$ (1)

where *f*_0_(*E*) = {1 + exp[(*E* – *E*_F_)/*k*_B_*T*]}^−1^ is the Fermi-Dirac distribution function, *E*_F_ is the Fermi energy in eV, *ρ*_c_(*E*) and *ρ*_v_(*E*) are, respectively, the electron density of states for the conduction band and the hole density of states for the valence band, *k*_B_ is the Boltzmann constant, and *T* is the temperature in kelvins. The calculations incorporate the Thomas-Fermi expression for the screening radius,

$$r_{s}^{2}=-\frac{4\pi e^{2}}{\varepsilon \varepsilon_{0}}\int_{-\infty }^{+\infty }\frac{df_{0}(E)}{dE}[\rho_{c}(E)-\rho_{v}(E)]dE,$$ (2)

and the charge neutrality condition

$$N_{I}=n–h,$$ (3)

to compute self-consistently the Fermi energy *E*_F_ and the screening radius *r*_s_ for given values of the ionized dopant concentration *N*_I_ and temperature *T*. The static dielectric constant is *ε* and the permittivity of free space is *ε*_0_. The ionized dopant concentration is positive for n-type material (donor ions) and negative for p-type material (acceptor ions). The results reported here are for uncompensated n-type material. The results for the screening radius *r*_s_ are not reported here because they are not needed to extract carrier concentrations from Raman measurements.

In this paper, we use the four-band model that has three conduction sub-bands centered at the Γ, L, and X symmetry points in the Brillouin zone and one equivalent valence band centered at the Γ symmetry point. We do not include the detailed nonparabolicity of the GaSb energy bands at Γ. Unlike GaAs, the GaSb conduction Γ, L, and X sub-band masses and energy spacings are such that for donor densities of technological interest, the conduction sub-band at L is the one that is most populated. The non-parabolicity of the conduction Γ sub-band in GaAs is discussed in Ref. [2]. If we were to use the Kane three level *k* · *p* model [2], which does not include the conduction sub-bands at L and X, we would be able to include the non-parabolicity of the conduction Γ sub-band. However, because the conduction Γ sub-band band in GaSb is not the dominant band for determining the Fermi energy, its non-parabolicity correction may not have a significant effect on the results given below and may lie within the uncertainties associated with the band masses quoted in the literature for GaSb.

The heavy hole mass *m*_hh_ and light hole mass *m*_lh_ for the two degenerate sub-bands at the top of the valence band are combined to give an effective mass

$$m_{v\Gamma }={\left({m_{\text{hh}}}^{3/2}+{m_{\text{lh}}}^{3/2}\right)}^{2/3},$$ (4)

for the valence topmost sub-band. The values of these parameters are given in Table 1.

The zero of energy is at the bottom of the conduction Γ sub-band. The bottoms of the conduction L and X sub-bands are, respectively, at *E*_cL_ and *E*_cX_. The top of the degenerate valence Γ sub-band is at −*E*_G_, where *E*_G_ is the bandgap of GaSb. The split-off valence sub-band at Γ due to spin-orbit coupling and the non-parabolicity factor of the conduction Γ sub-band are neglected. The probabilities for typical carriers in equilibrium to occupy appreciably these states are low. This means that the Fermi energies should be sufficiently above the valence sub-band maximum at Γ. Placing exact limits on the Fermi energies for which the four-band model is valid would be tenuous, because knowledge of how the various sub-bands move relative to one another due to the dopant concentrations considered here and due to many body effects is not adequate.

The general expression [3] for the temperature dependence of conduction sub-band minima relative to the top of the valence band at Γ is

$$E_{i}=E_{i0}-\left[A_{i}T^{2}/\left(T+B_{i}\right)\right]$$ (5)

in units of eV, where *i* = Γ, L, or X. The values for the coefficients *E_i_*_0_, *A_i_*, and *B_i_* are listed in Table 2.

The general expression for the parabolic densities of states for electrons and holes per band extrema and per spin direction is given by

$$\rho (E)=\frac{N_{e}4\pi V\sqrt{E}}{\left(8\pi^{3}\right){\left(\hbar^{2}/2m^{*}m_{0}\right)}^{3/2}},$$ (6)

where *N*_e_ is the number of equivalent ellipsoids in the first Brillouin zone, the volume of the unit cell is *V* = *a*_L_^3^, *a*_L_ is the lattice constant, m* is one of the effective masses listed in Table 1 for the appropriate band extrema, and *m*_0_ is the free electron mass. Because eight permutations of the wave vector in the (111) direction exist, there are eight L sub-band ellipsoids with centers located near the boundary of the first Brillouin zone. Also, because six permutations of the wave vector in the (100) direction exist, there are six X sub-band ellipsoids with centers located near the boundary of the first Brillouin zone. Since about half of each ellipsoid is in the neighboring zone, the number of equivalent sub-bands *N*_cL_ for the L sub-band is four and the number of equivalent sub-bands *N*_cX_ for the X sub-band is three.

In terms of a four-band model for room temperature n-type GaSb, the total density of states *ρ*_c_(*E*) for the majority carrier electrons in n-type GaSb then becomes

$$\rho_{c}\left(E\right)=\rho_{c\Gamma }\left(E\right)+\rho_{\text{cL}}\left(E\right)+\rho_{\text{cX}}\left(E\right),$$ (7)

where *ρ*_cΓ_(*E*), *ρ*_cL_(*E*), and *ρ*_cX_(*E*) are the sub-band densities of states for the conduction Γ, L, and X sub-bands with effective masses of *m*_cΓ_, *m*_cL_, and *m*_cX_, respectively. The density of states for the minority carrier holes is

$$\rho_{v}\left(E\right)=\rho_{v\Gamma }\left(E\right)$$ (8)

with an effective mass of *m*_vΓ_.

## 3. Results

**Fig. 1:**
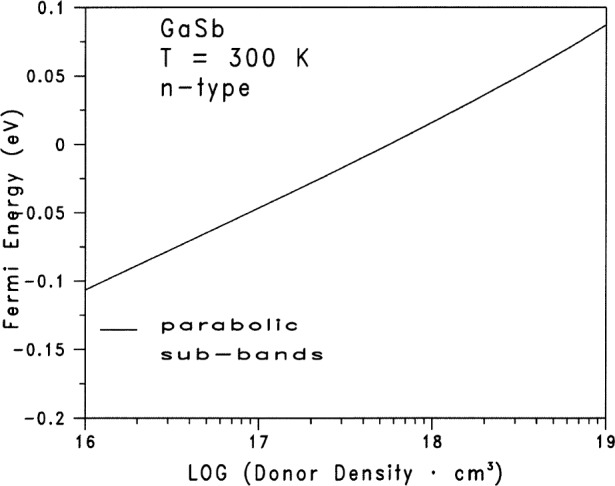
The calculated Fermi energy for n-type GaSb at 300 K as a function of the donor density. The Fermi energy is relative to the majority conduction band edge at the *Γ* symmetry point in the first Brillouin zone.

**Fig. 2:**
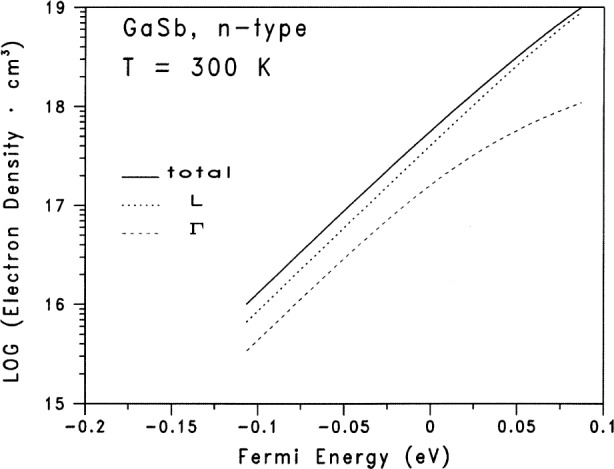
The calculated electron densities in the conduction sub-bands at *Γ* and *L* and the total electron density as functions of the Fermi energy. The Fermi energy is relative to the majority conduction band edge at the *Γ* symmetry point in the first Brillouin zone.

Tables 1 and 2 contain the input parameters for the calculations of the Fermi energy as a function of the dopant donor density. We solve self-consistently, by means of an iterative procedure, Eq. (3) with Eqs. (6), (7) and (8). The independent variable is the temperature *T*. The Fermi energy is varied for a given temperature until Eq. (3) is satisfied. Figure 1 presents the calculated data graphically for 28 values of donor densities between 10^16^ cm^−3^ and 10^19^ cm^−3^. Figure 2 gives the electron densities in the conduction sub-bands at Γ and L and the total electron density as functions of the Fermi energy. Figure 2 does not show the electron density in the conduction sub-band at X, because it is less than 10^−3^ times the total electron density. Because *m*_cΓ_ << *m*_cL_ and *E*_cL_ is much closer to *E*_cΓ_ than it is to *E*_cX_, the electron density in the conduction L sub-band exceeds the electron density in the conduction Γ sub-band at room temperature. The solid curve in Fig. 2 is the same curve as given in Fig. 1. Figure 2 shows that the majority of electrons is in the conduction L sub-band and that the density of electrons in the L sub-band approaches the total density of electrons as the donor density approaches 10^19^ cm^−3^. Hence, even though GaSb is intrinsically a direct semiconductor, the results from Fig. 2 suggest that electrons for n-type GaSb in the vicinity of the Fermi surface will behave as though they have many characteristics of electrons in an indirect semiconductor.

For illustrative purposes, we give here only the results for fitting the logarithm to the base 10 of the total electron density *n* in units of cm^−3^ to a polynomial in *E*_F_, namely,

$$\log_{10}(n\;{\text{cm}}^{-3})=a_{0}+a_{1}E_{F}+a_{2}E_{F}^{2}+\ldots +a_{1}E_{F}^{1}\ldots .$$ (9)

The analytic fits for the electron densities in the Γ, L, and X sub-bands are available by sending an email to herbert.bennett@nist.gov. During the fitting analyses, we rely substantially on graphics and keep the number of fitting parameters to a minimum, subject to the constraint that the residual standard deviation *S*_res_ is acceptably small, i.e., *S*_res_ ≤ 0.01. The standard deviation is a measure of the “average” error in a fitted model and thereby is a metric for assessing the quality of the fit. A smaller *S*_res_ indicates a better fit. The residual standard deviation for a model *Y^f^* = *f* (*Z*) is

$$S_{\text{res}}=\sqrt{[\sum_{j=1}^{N}{\left(Y_{j}-{\bar{Y}}_{j}^{f}\right)}^{2}/(N-P)]},$$ (10)

where *Y_j_* are the calculated data values, the ${\bar{Y}}_{j}^{f}$ are the predicted values from the fitted model, *N* is the total number of data points (here *N* = 28), and *P* is the total number of parameters to be fitted in the model. We use the NIST-developed DATAPLOT [4] software for both the exploratory graphics and for the statistical analyses.

Table 3 gives the four fitting parameters for the cubic *l* = 3 polynomial fit to the log_10_(*n*) as shown in Eq. (9) and the associated residual standard deviation *S*_res_ = 0.0066. In general, the values of *S*_res_ decrease monotonically with increasing number *l* of terms in the polynomial given in Eq. (9). But, care must be taken to avoid fitting noise in data sets. The general guideline for many data sets is that when the absolute value of the ratio *R* of the estimated parameter value divided by its estimated standard deviation is less than about 2, then the rate of decrease in *S*_res_ with increasing *l* tends to decrease. For the data given in Fig. 1, when *l* = 2 or P = 3, *S*_res_ = 0.0644; when *l* = 3 or *P* = 4, *S*_res_ = 0.0066; and when *l* = 4 or *P* = 5, *S*_res_ = 0.0063. Because the change in values of *S*_res_ between *l* = 3 and *l* = 4 is not significant, we use the fitting parameters for the cubic *l* = 3 case in this paper. Also, the ratio *R* for the parameter *a*_4_ when *l* = 4 is −1.917, and such a value for *R* means that proceeding with higher *l* values probably is not warranted. A figure that compares the calculated total electron density as a function of the Fermi energy with the fitting results from Eq. (9) for a cubic polynomial is not given because the two curves lie on top of one another within the line widths of each curve. Fits to the calculated electron densities for each of the conduction sub-bands are available from the author upon request. Also, since the screening radii for the carriers from Eq. (2) are not needed when interpreting the proposed measurements considered here, the corresponding screening radii are not presented in this paper.

## 4. Conclusions

The results from Sec. 3 are consistent with the findings of experimental work reported in the literature such as Refs. [5] and [6]. Interpreting experiments for GaSb requires at least a three-band model and under some conditions may require a four-band model. And finally, even though GaSb is intrinsically a direct semiconductor, our results show that electrons for n-type GaSb in the vicinity of the Fermi surface will have some characteristics that are similar to those for electrons in an indirect semiconductor.

## Figures and Tables

**Table 1:** Input parameters for intrinsic zinc blende GaSb at 300 K. The energies of the extrema of the conduction and valence sub-bands are referenced to the bottom of the conduction sub-band at the Γ symmetry point in the Brillouin zone of the reciprocal lattice space. The mass of the free electron is *m*_0_. These data are from Ref. [3]

Parameter	Symbol	Value	Units
Lattice constant	*a*_L_	6.09593 × 10^−8^	cm
Dielectric constant in vacuum	*ε*	8.854 × 10^−12^	F/m
Static dielectric constant	*ε*_0_	15.7	
Bandgap	*E*_G_ = |*E*_vΓ_|	0.726	eV
Bottom of the conduction L sub-band	*E*_cL_	0.084	eV
Bottom of the conduction X sub-band	*E*_vX_	0.31	eV
Top of the degenerate valence Γ sub-band	−*E*_vΓ_	−0.726	eV
Spin-orbit splitting	*E*_so_	0.80	eV
Top of the split-off (spin-orbit splitting) valence Γ sub-band	−*E*_soΓ_ = − *E*_vΓ_ − *E*_so_	−1.526	eV
Effective mass of conduction Γ sub-band	*m*_cΓ_	0.041	*m*_0_
Transverse *L* sub-band mass	*m*_tL_	0.11	*m*_0_
Longitudinal *L* sub-band mass	*m*_lL_	0.95	*m*_0_
Effective mass of conduction *L* sub-band	*m*_cL_ = (*m*_lL_ *m*_tL_^2^)^1/3^	0.226	*m*_0_
Transverse X sub-band mass	*m*_tX_	0.22	*m*_0_
Longitudinal X sub-band mass	*m*_lX_	1.51	*m*_0_
Effective mass of conduction X sub-band	*m*_cX_ = (*m*_lX_ *m*_tX_^2^)^1/3^	0.418	*m*_0_
Light hole mass of degenerate valence Γ sub-band	*m*_lh_	0.05	*m*_0_
Heavy hole mass of degenerate valence Γ sub-band	*m*_hh_	0.4	*m*_0_
Effective mass of degenerate valence Γ sub-band	*m*_vΓ_	0.41	*m*_0_
Splitoff band mass of the valence sub-band at Γ	*m*_so_	0.14	*m*_0_
Number of equivalent conduction L sub-bands	*N*_cL_	4	
Number of equivalent conduction X sub-bands	*N*_cX_	3	

**Table 2:** Coefficients for the temperature dependence of the conduction band extrema that are used in Eq. (5). These data are from Ref. [3]

Parameter	Symbol	Value	Units
Γ sub-band	*E*_Γ0_	0.813	eV
Γ sub-band	*A*_Γ_	3.78 × 10^−4^	eV/K
Γ sub-band	*B*_Γ_	94.	K
L sub-band	*E*_L0_	0.902	eV
L sub-band	*A*_L_	3.97 × 10^−4^	eV/K
L sub-band	*B*_L_	94.	K
X sub-band	*E*_X0_	1.142	eV
X sub-band	*A*_X_	4.75 × 10^−4^	eV/K
X sub-band	*B*_X_	94.	K

**Table 3:** The four fitting parameters for a cubic polynomial fit of the theoretical calculations for the total electron density in n-type, zinc blende GaSb at 300 K as a function of the Fermi energy relative to the bottom of the conduction Γ sub-band. The ratio is the estimated value divided by the estimated standard deviation. The residual standard deviation is *S*_res_ = 0.0066

Fitting parameter	Estimated value	Estimated standard deviation	Units	Ratio
*a*_0_	17.7504	0.1774 × 10^−3^		1.001 × 10^5^
*a*^1^	15.6775	0.5416 × 10^−2^	eV^−1^	2.895 × 10^3^
*a*^2^	−11.4745	0.4723 × 10^−1^	eV^−2^	−2.43 × 10^2^
*a*^3^	−41.3848	0.8535	eV^−3^	−4.849 × 10^1^
